# Spatiotemporal Locality-Aware Adaptive Hybrid Optoelectronic Interconnect for Reconfigurable Array Processors

**DOI:** 10.3390/s26092871

**Published:** 2026-05-04

**Authors:** Bowen Yang, Yong Li, Rui Shan, Junyong Deng, Yu Feng

**Affiliations:** 1 School of Electronics and Information, Northwestern Polytechnical University, Xi’an 710072, China; 2 School of Electronic Engineering, Xi’an University of Posts & Telecommunications, Xi’an 710121, China

**Keywords:** hybrid optoelectronic NoCs, spatiotemporal locality, adaptive routing, medium selection, cross-layer congestion, reconfigurable computing

## Abstract

As data-intensive applications continue to scale reconfigurable array processors (RAPs), electrical networks-on-chip (NoCs) are increasingly constrained by energy-delay bottlenecks due to RC-delay constraints. Hybrid optoelectronic NoCs (HONoCs) suffer from a fundamental medium-selection dilemma: optical circuit switching incurs microsecond-scale setup overheads for long flows, whereas static distance thresholds fail to capture the spatiotemporal heterogeneity of traffic, causing wavelength waste for bursty flows and congestion diffusion under non-stationary loads. This paper presents an adaptive switching framework that is aware of spatiotemporal locality. We introduce the Temporal-Spatial Locality Index (TSLI) to classify flows into Electrophilic (EF), Photophilic (PF), and Hybrid-sensitive (HF) categories, and propose Cross-layer Congestion Entropy (CCE) to unify electrical and optical resource states. Based on these metrics, an Adaptive Medium Selection State Machine (AMSSM) dynamically switches among Electro-Dominant (EDM), Electro-Optical Synergistic (EOSM), and Optical-Dominant (ODM) modes, while a Weighted Multi-dimensional Medium Matching (WMMM) algorithm performs fine-grained channel selection. A Predictive Optical Path Provisioning (POPP) mechanism further amortizes setup latencies via trend-aware pre-establishment. Evaluation on an 8 × 8 mesh HONoCs demonstrates 22% higher saturation throughput, 38% lower energy-delay product (EDP), and 57% reduction in average latency under non-stationary traffic, compared to static thresholds. The proposed mechanisms provide a theoretical foundation and engineering paradigm for efficient on-chip interconnects.

## 1. Introduction

The proliferation of data-intensive computational workloads—including deep neural network (DNN) training/inference, high-efficiency video coding (HEVC), and large-scale scientific simulations—has catalyzed the development of reconfigurable array processors (RAPs) as a promising paradigm to overcome the inflexibility of traditional fixed-function architectures. RAPs, characterized by arrays of processing element groups (PEGs) interconnected via programmable switching fabrics, offer hardware-level adaptability that enables algorithm-to-hardware mapping for improved energy efficiency and performance [1,2]. However, as semiconductor technology nodes advance below 14 nm, the communication substrate of these systems has emerged as a critical bottleneck. In contemporary RAPs implementing HEVC encoding or CNN inference, data movement between PEGs can consume over 50% of total execution cycles and contribute significantly to overall energy consumption [3,4].

Traditional electrical Networks-on-Chip (NoCs) face fundamental physical limitations that constrain their scalability. The RC delay of electrical interconnects increases linearly with transmission distance, while dynamic energy consumption scales quadratically with distance due to capacitive charging of long wires [5,6,7]. Furthermore, at high clock frequencies, the skin effect increases effective resistance, and crosstalk between adjacent wires exacerbates signal integrity issues. These effects collectively create a “communication energy wall” that limits the practical size of purely electrical many-core systems [8,9].

Silicon photonics presents a compelling alternative for on-chip communication, leveraging photonic carrier waves that propagate at effective velocities approaching $2\times 10^{8}$ m/s with negligible attenuation and distance-independent transmission energy. Optical interconnects based on micro-ring resonators (MRRs) and silicon modulators can achieve bandwidth densities exceeding 10× that of electrical counterparts [10,11]. However, practical deployment of photonic interconnects in RAPs introduces the medium selection dilemma: Optical Circuit Switching (OCS), the predominant paradigm for on-chip photonics due to the lack of practical optical buffering, requires microsecond-scale setup times ($t_{setup}\approx 1$–2 μs) encompassing MRR thermal tuning (typically 1–2 μs for silicon thermo-optic effects), wavelength allocation arbitration, and transceiver synchronization [12,13].

This setup overhead creates an asymmetric cost structure: optical transmission is efficient only when flow duration $T_{dur}\gg t_{setup}$, allowing the setup energy $E_{setup}$ to be amortized over many bits. For short bursty flows (SBFs), forced optical transmission results in negative energy efficiency and latency inflation. Conversely, long streaming flows (LSFs) misclassified to electrical paths cause buffer accumulation, head-of-line blocking, and congestion diffusion across the electrical fabric [14,15,16].

Existing HONoCs designs predominantly employ static distance thresholds (e.g., “use optical if Manhattan distance h≥2 hops”) or simple latency comparisons [17,18]. These approaches suffer from three critical limitations: (1) Temporal blindness—they ignore flow duration and burstiness, treating a 10-flit control packet identically to a 1000-flit data stream if both traverse the same distance; (2) Spatial rigidity—static thresholds cannot adapt to dynamic network congestion or wavelength availability; (3) Layer fragmentation—electrical buffer occupancy and optical wavelength utilization are monitored independently, leading to globally suboptimal decisions such as “optical resources idle while electrical buffers overflow” or “wavelengths wasted on insufficient flows.”

## 2. Problem Formulation

Consider a hybrid optoelectronic NoCs comprising N nodes arranged in a 2D mesh topology, where each node i maintains both electrical ports with buffer capacity $B_{max}$ and optical ports supporting W wavelengths via WDM [19,20,21]. A data flow F is defined as a sequence of packets originating at source s and terminating at destination d, characterized by the following: (i) Spatial span—$H(s,d)=|x_{s}-x_{d}|+|y_{s}-y_{d}|$ (Manhattan distance) [22,23]; (ii) Temporal profile—Start time $t_{start}$, observed duration $T_{dur}$, and mean arrival rate $\lambda_{avg}$ (flits/cycle); (iii) Load characteristics—total payload $L_{payload}$ and burstiness $\sigma_{traffic}$.

The medium selection decision m(F)∈{0,1} (0: electrical, 1: optical) must minimize a composite cost function:

$$\underset{m(F)}{min}C(F,m)=\alpha \frac{T(F,m)}{T_{ref}}+\beta \frac{E(F,m)}{E_{ref}}+\gamma \frac{R(F,m)}{R_{ref}}$$

subject to the following: 1. Optical resource constraint—$\sum_{F\in F_{opt}}w(F)\leq W_{total}$ (wavelength conservation); 2. Electrical capacity constraint—$\rho_{elect}\leq \rho_{th}$ (congestion avoidance); 3. Setup amortization constraint—if m(F)=1, then $T_{dur}>t_{setup}$ must hold in expectation where T(F,m) represents end-to-end latency, E(F,m) represents energy consumption, R(F,m) represents resource occupancy, and α+β+γ=1 are application-dependent weights.

## 3. Proposed Approach and Contributions

To address the medium selection dilemma while respecting the stringent resource constraints of on-chip routers (KB-level storage, nanosecond-scale control latency), this paper proposes a spatiotemporal locality-aware adaptive framework with the following contributions.

First, we establish a rigorous theoretical foundation by introducing the Temporal-Spatial Locality Index (TSLI), a dimensionless metric that unifies spatial span and temporal persistence:

(1) $$\Lambda (F)=\frac{H(s,d)}{T_{dur}\cdot \lambda_{avg}}$$

where $H\left(s,d\right)$ denotes the Manhattan distance between source and destination nodes, $T_{dur}$ represents the observed flow duration in clock cycles, and $\lambda_{avg}$ is the mean flit arrival rate (flits/cycle). Physically, Λ(F) captures the spatial diffusion coefficient per flit.

From an information-theoretic perspective, Λ(F) quantifies the entropy of flow distribution in the spatiotemporal domain. Low Λ indicates concentrated, persistent flows suitable for optical transport, and high Λ indicates dispersed, bursty flows suitable for electrical transport. We derive classification thresholds $\tau_{low}$ and $\tau_{high}$ that dynamically adapt based on cross-layer congestion states, enabling refined categorization into Electrophilic Flows (EF), Photophilic Flows (PF), and Hybrid-sensitive Flows (HF).

Second, we propose Cross-layer Congestion Entropy (CCE), a unified metric fusing electrical buffer distribution entropy $H_{elect}$ and optical wavelength utilization entropy $H_{opt}$:

(2) $$CCE=\omega_{elect}\cdot \frac{H_{elect}}{H_{elect}^{max}}+\omega_{opt}\cdot \frac{H_{opt}}{H_{opt}^{max}}+\omega_{load}\cdot \frac{\rho_{elect}+\rho_{opt}}{2}$$

This resolves the fragmentation between electrical and optical resource monitoring, providing a global view of heterogeneous resource competition.

Third, we design the Adaptive Medium Selection State Machine (AMSSM) featuring three operating modes—Electro-Dominant Mode (EDM), Electro-Optical Synergistic Mode (EOSM), and Optical-Dominant Mode (ODM)—with hysteresis-based anti-oscillation mechanisms and dynamic threshold adjustment. Within EOSM, the Weighted Multi-dimensional Medium Matching (WMMM) algorithm evaluates candidates across four dimensions: Transmission Energy Efficiency (TEE), Link Availability (LA), Medium Affinity Match (MAM), and Congestion Evasion Weight (CEW), implementing a 5-cycle hardware-friendly pipeline.

Fourth, we introduce Predictive Optical Path Provisioning (POPP), which leverages second-order trend analysis of $\rho_{o}$ (photophilic flow ratio) to pre-establish optical paths before data arrival, transforming OCS from reactive to predictive. An Optical Escape Mechanism provides emergency congestion relief when electrical buffers exceed critical thresholds.

Extensive evaluation demonstrates that the proposed mechanisms achieve 22% improvement in saturation throughput, 38% reduction in Energy-Delay Product (EDP), and 57% decrease in average latency compared to static threshold baselines under non-stationary traffic, while requiring only 1.3 KB parameter storage and 12-cycle inference latency for the lightweight prediction engine.

## 4. Background and Related Work

### 4.1. Hybrid Optoelectronic NoCs Architectures

The integration of photonics with CMOS electronics for on-chip communication has evolved from simple point-to-point links to sophisticated network architectures. Early work by Luo et al. [24] and Agarwal et al. [25] established the feasibility of optical NoCs using micro-ring resonator-based switching. Subsequent architectures such as Firefly [26], TAONoC [27], and WRONoC [28] adopted hierarchical approaches where electrical meshes handle short-range, bursty traffic while optical global links manage long-range, high-bandwidth communication.

However, these architectures predominantly employ static allocation policies. For instance, Firefly uses electrical networks for intra-cluster communication and optical networks for inter-cluster traffic based on fixed distance thresholds. Such static partitioning fails to account for dynamic traffic variations, leading to suboptimal resource utilization. Recent work by Yao et al. [29] explored dynamic wavelength allocation, but relied on simple latency comparisons rather than systematic flow characterization.

### 4.2. Traffic Management in Heterogeneous Interconnects

The management of heterogeneous resources in NoCs has been approached through various lenses. Conventional adaptive routing in electrical NoCs uses local congestion information (buffer occupancy, crossbar contention) to dynamically select paths [30,31]. However, these techniques are confined to single-layer optimization and do not address the electo-optical boundary.

Machine learning approaches have been proposed for datacenter network traffic prediction, utilizing LSTM or Transformer architectures to capture long-range dependencies [32]. However, these models typically require MB-scale parameter storage and hundreds of cycles for inference, violating the microsecond-scale timing constraints of on-chip router control planes. Lightweight alternatives such as Exponentially Weighted Moving Average (EWMA) suffer from significant prediction lag (Mean Absolute Percentage Error > 38%) under non-stationary traffic patterns [14], motivating the need for structured spatiotemporal models that balance accuracy and hardware feasibility.

### 4.3. Theoretical Foundations

From an information-theoretic perspective, network traffic can be modeled as stochastic processes with spatiotemporal correlations. The entropy rate of a traffic source characterizes its predictability and burstiness. However, existing NoCs literature lacks a principled framework connecting these information-theoretic properties to physical medium selection. Our work bridges this gap by interpreting TSLI as a measure of spatiotemporal entropy, thereby grounding medium selection in fundamental information theory.

## 5. Spatiotemporal Locality and Cross-Layer Congestion

### 5.1. Temporal-Spatial Locality Index: Theoretical Foundation

The Temporal-Spatial Locality Index (TSLI) is derived from first principles of flow transport efficiency. Consider a flow F traversing distance H with duration $T_{dur}$ and average rate $\lambda_{avg}$. The total number of flits transmitted is $N_{flits}=\lambda_{avg}\cdot T_{dur}$. The efficiency of optical transmission depends on the ratio of setup overhead to useful transmission time:

(3) $$\eta_{opt}=\frac{T_{dur}}{T_{dur}+t_{setup}}\cdot \frac{1}{E_{OEO}+E_{tuning}/N_{flits}}$$

For optical transmission to be preferable to electrical transmission (which scales as $E_{elect}\propto H$), the break-even condition requires the following:

(4) $$\frac{H}{T_{dur}\cdot \lambda_{avg}}<\tau_{critical}$$

where $\tau_{critical}$ is a technology-dependent threshold incorporating $E_{OEO}$, $E_{tuning}$, and $t_{setup}$. This motivates the definition as follows:

(5) $$\Lambda (F)=\frac{H}{T_{dur}\cdot \lambda_{avg}}$$

Information-Theoretic Interpretation: From an information-theoretic perspective, consider the spatiotemporal domain as a two-dimensional probability space. The spatial entropy $H_{spatial}=-\sum p(d)logp(d)$ characterizes the distribution of destinations, while temporal entropy $H_{temporal}$ characterizes inter-arrival patterns. For point-to-point flows, Λ(F) is inversely proportional to the product of spatial span and temporal persistence, serving as a proxy for the joint spatiotemporal entropy. Low entropy (concentrated in space and time) implies predictable, stream-like behavior suitable for circuit-switched optical paths; high entropy implies dispersed, unpredictable behavior suitable for packet-switched electrical paths.

Dynamic Threshold Adaptation: Static thresholds $\tau_{low}$ and $\tau_{high}$ cannot adapt to network load variations. We derive dynamic thresholds based on cross-layer congestion:

(6) $$\tau_{high}(t)=\tau_{high}^{base}\cdot (1+\eta \cdot \rho_{elect}(t))$$

(7) $$\tau_{low}(t)=\tau_{low}^{base}\cdot (1-\mu \cdot \rho_{opt}(t))$$

where $\rho_{elect}$ and $\rho_{opt}$ are the mean utilization rates of electrical and optical resources, respectively, and η,μ∈[0.1,0.3] are sensitivity coefficients. When electrical congestion increases ($\rho_{elect}↑$), $\tau_{high}$ increases, effectively lowering the barrier for EF flows to migrate to optical paths (electrophilic relaxation). Conversely, when optical resources become scarce ($\rho_{opt}↑$), $\tau_{low}$ increases, preventing premature optical allocation.

Medium Affinity Classification: Based on Λ(F) and dynamic thresholds, flows are classified into three categories.

1. Electrophilic Flow (EF): $\Lambda (F)>\tau_{high}$

○ Characteristics—High spatial dispersion or short temporal persistence
○ Physical interpretation—Spatially global but temporally transient, or spatially local with bursty patterns
○ Medium selection—Prefer electrical paths to avoid OEO conversion overhead and wavelength fragmentation.

2. Photophilic Flow (PF): $\Lambda (F)<\tau_{low}$

○ Characteristics—Sustained transmission over long distances
○ Physical interpretation—Spatially global and temporally persistent
○ Medium selection—Prefer optical paths to amortize setup costs over long transmissions.

3. Hybrid-sensitive Flow (HF): $\tau_{low}\leq \Lambda (F)\leq \tau_{high}$

○ Characteristics—Intermediate spatiotemporal characteristics
○ Physical interpretation—Moderate distance and duration where medium choice depends on instantaneous resource availability
○ Medium selection—Require comprehensive multi-dimensional evaluation via WMMM.

### 5.2. Cross-Layer Congestion Entropy

Traditional NoCs monitoring treats electrical and optical layers independently, leading to fragmented decision-making. The electrical layer reports buffer occupancy $\{b_{j}{\}}_{j=1}^{k}$ while the optical layer reports wavelength usage $\{q_{w}{\}}_{w=1}^{W}$. Independent optimization leads to local optima: an electrical port may be congested ($b_{j}\approx B_{max}$) while optical wavelengths remain idle, or wavelengths may be allocated to marginal flows while electrical buffers overflow.

Electrical Layer Entropy: For a router with k output ports, the normalized occupancy distribution is defined as

(8) $$p_{j}=\frac{b_{j}}{\sum_{i=1}^{k}b_{i}+\epsilon }$$

The Shannon entropy of this distribution measures load balance:

(9) $$H_{elect}=-\sum_{j=1}^{k}p_{j}{log}_{2}(p_{j}+\epsilon )$$

$H_{elect}=0$ indicates absolute hotspot (all traffic concentrated on one port), while $H_{elect}={log}_{2}k$ indicates perfect uniform distribution. However, $H_{elect}$ alone is insufficient: a uniform distribution at 95% capacity is still congested. Thus, we incorporate the mean utilization $\rho_{elect}=\frac{1}{k}\sum b_{j}/B_{max}$.

Optical Layer Entropy: Similarly, for wavelength utilization rates $\left\{q_{w}\right\}$

(10) $$H_{opt}=-\sum_{w=1}^{W}\frac{q_{w}}{\sum q_{w}}{log}_{2}\left(\frac{q_{w}}{\sum q_{w}}+\epsilon \right)$$

Low $H_{opt}$ indicates wavelength concentration (hotspots on specific wavelengths), while high $H_{opt}$ indicates fragmentation (wavelengths scattered across many ports, potentially causing blocking for wide-spectrum flows).

Unified Cross-Layer Metric: We propose the Cross-layer Congestion Entropy (CCE) as a weighted combination:

(11) $$CCE=\omega_{elect}\cdot \frac{H_{elect}}{H_{elect}^{max}}+\omega_{opt}\cdot \frac{H_{opt}}{H_{opt}^{max}}+\omega_{load}\cdot \frac{\rho_{elect}+\rho_{opt}}{2}$$

where $H^{max}={log}_{2}(\mathrm{ports})$ or ${log}_{2}(W)$ normalizes entropy to [0,1], and weights satisfy $\omega_{elect}+\omega_{opt}+\omega_{load}=1$ (typically 0.4,0.4,0.2).

The three congestion zones derived from CCE guide system behavior are as follows: Low Congestion (CCE<0.3)—Both layers have balanced, low utilization. The system defaults to simple heuristics (prefer electrical for EF, optical for PF) to minimize control overhead; Medium Congestion (0.3≤CCE≤0.7)—One or both layers exhibit imbalance. The system activates full WMMM evaluation for HF flows to perform load balancing; High Congestion (CCE>0.7)—Critical resource scarcity. The system triggers optical escape via reserved emergency wavelengths for electrical hotspots; if the reserved optical capacity is also exhausted, the system falls back to credit-based electrical backpressure to upstream nodes, stalling injection until local congestion clears.

## 6. Adaptive Control Mechanisms

### 6.1. Adaptive Medium Selection State Machine (AMSSM)

The AMSSM implements a hysteresis-based finite state machine operating at observation window granularity $T_{window}=1024$ cycles. The state space S={EDM,EOSM,ODM} transitions are based on the photophilic flow ratio $\rho_{o}$ (fraction of traffic classified as PF) and the cross-layer congestion entropy CCE.

**Figure 1:**
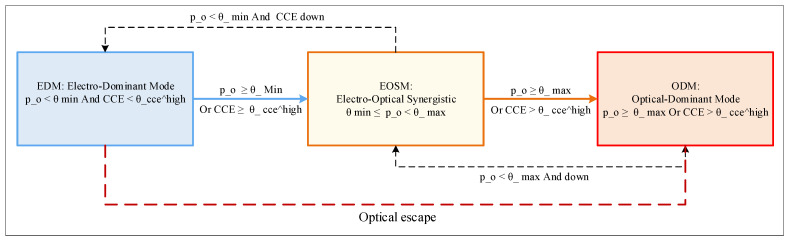
State transition diagram of the AMSSM.

State Definitions and Invariants shown in Figure 1:

Mode 1: Electro-Dominant Mode (EDM). Entry condition—$\rho_{o}<\theta_{min}$ AND $CCE<\theta_{high}$. Invariant: P(electrical selection)>0.9 for all flow types. Strategy—EF flows: Immediate electrical assignment. HF flows: Electrical assignment unless destination port occupancy $>3/4B_{max}$. PF flows: Electrical assignment unless $T_{dur}>5\cdot t_{setup}$ (rare override). Optical layer: Placed in standby; laser biasing reduced to save static power. Emergency exception: Optical escape triggered if any electrical port maintains $b_{j}>3/4B_{max}$ for duration $t_{emergency}>4$ cycles.

Mode 2: Electro-Optical Synergistic Mode (EOSM). Entry condition—$\left(\theta_{min}\leq \rho_{o}<\theta_{max}\right)$ OR $\left(\theta_{low}\leq CCE<\theta_{high}\right).$ Invariant: Balanced utilization, both media actively employed. Strategy—EF flows: Electrical (default). PF flows: Optical (default) with POPP pre-establishment. HF flows: Full WMMM evaluation (5-cycle pipeline). Optical layer: Active with aggressive pre-provisioning for predicted PF flows.

Mode 3: Optical-Dominant Mode (ODM). Entry condition—$\rho_{o}\geq \theta_{max}$ OR ($CCE>\theta_{high}$ AND $H_{elect}\ll H_{opt}$). Invariant: P(optical selection)>0.8 for non-EF flows. Strategy—EF flows: Electrical (only if optical wavelengths exhausted). HF/PF flows: Optical with extended hold times. Optical layer: Maximum utilization; electrical layer reserved for control and emergency.

Anti-Oscillation Mechanism: To prevent state thrashing (rapid EDM↔EOSM↔ODM transitions) under fluctuating traffic, we implement a saturation counter requiring n=3 consecutive windows satisfying transition conditions before state change. The transition logic incorporates hysteresis bands. EDM→EOSM: Requires $\rho_{o}\geq \theta_{min}$ AND $\Delta \rho_{o}>0$ (rising trend). EOSM→ODM: Requires $\rho_{o}\geq \theta_{max}$ AND rising CCE. ODM→EOSM: Requires $\rho_{o}<\theta_{max}$ AND falling trend ($\Delta \rho_{o}<0$) for 3 windows OR $W_{avail}<10\%$ (optical exhaustion). EOSM→EDM: Requires $\rho_{o}<\theta_{min}$ AND $CCE<\theta_{low}$ for 3 windows

Dynamic Threshold Adjustment: Thresholds $\theta_{min},\theta_{max}$ adapt based on CCE:

(12) $$\theta_{min}(t)=\theta_{min}^{base}\cdot (1+\sigma \cdot CCE(t))$$

where σ=0.2 controls sensitivity. As congestion increases, the system lowers barriers to optical migration to relieve electrical pressure.

### 6.2. Weighted Multi-Dimensional Medium Matching (WMMM)

When operating in EOSM (or for HF flows in any mode), the system requires fine-grained selection among candidate channels. WMMM evaluates each candidate c (electrical port or optical wavelength) across four dimensions:

Dimension 1: Transmission Energy Efficiency (TEE). The energy efficiency of transmitting flow F via channel c is a follows:

(13) $$TEE_{c}=\left\{\begin{array}{ll} \frac{1}{E_{elect}(h)}=\frac{1}{E_{switch}+h\cdot E_{wire}} & \mathrm{if}\;c\in C_{elect}\\ \frac{{\hat{T}}_{dur}}{E_{OEO}\cdot L_{payload}+E_{tuning}+P_{laser}\cdot {\hat{T}}_{dur}} & \mathrm{if}\;c\in C_{opt} \end{array}\right.$$

For optical channels, the numerator ${\hat{T}}_{dur}$ (predicted flow duration) ensures that short flows are penalized by the fixed setup costs $E_{tuning}$ and $P_{laser}\cdot {\hat{T}}_{dur}$.

Dimension 2: Link Availability (LA). The immediate capacity of the channel is as follows:

(14) $$LA_{elect}=1-\frac{b_{current}}{B_{max}}$$

(15) $$LA_{opt}=\frac{W_{free}}{W_{total}}$$

For optical channels, $W_{free}$ counts wavelengths are available on the specific path, accounting for wavelength continuity constraints.

Dimension 3: Medium Affinity Match (MAM). Alignment between flow characteristics and medium properties is as follows:

(16) $$MAM_{c}=\left\{\begin{array}{ll} exp\left(-\frac{\Lambda (F)}{\tau_{high}}\right) & \mathrm{if}c\in C_{elect}\\ exp\left(-\frac{\tau_{low}}{\Lambda (F)}\right)\cdot I({\hat{T}}_{dur}>t_{setup}) & \mathrm{if}c\in C_{opt} \end{array}\right.$$

The indicator function $I({\hat{T}}_{dur}>t_{setup})$ ensures optical channels are only considered if the predicted duration amortizes the setup time. The exponential form provides smooth differentiation near thresholds.

Dimension 4: Congestion Evasion Weight (CEW). The dynamic penalty based on layer-specific congestion is the following:

(17) $$CEW_{c}=\left\{\begin{array}{ll} 1-\omega_{elect}\cdot \frac{H_{elect}}{H_{elect}^{max}} & \mathrm{if}c\in C_{elect}\\ 1-\omega_{opt}\cdot \frac{H_{opt}}{H_{opt}^{max}} & \mathrm{if}c\in C_{opt} \end{array}\right.$$

When a layer exhibits high entropy (imbalanced load), CEW reduces the score for that layer, steering traffic toward the less congested medium.

Composite Scoring and Selection. The final score is a weighted sum as follows:

(18) $$S_{c}=\lambda_{1}\frac{TEE_{c}}{TEE_{max}}+\lambda_{2}LA_{c}+\lambda_{3}MAM_{c}+\lambda_{4}CEW_{c}$$

with $\sum \lambda_{i}=1$. Default weights (0.3,0.3,0.25,0.15) balance energy efficiency, immediacy, affinity, and load balancing. The channel with maximum $S_{c}$ is selected; ties favor electrical paths to conserve optical resources.

Hardware Pipeline: WMMM is implemented as a 4-stage pipeline. Stage 1: Parameter fetch (TSLI, CCE, buffer states)—1 cycle. Stage 2: Parallel TEE/LA/MAM/CEW computation—2 cycles (exponential approximations via LUTs). Stage 3: Weighted accumulation—1 cycle (DSP48 MACs). Stage 4: Comparison tree—1 cycle (finding argmax). Total latency: 5 cycles @ 250 MHz = 20 ns, fitting within the router microarchitecture pipeline.

### 6.3. Predictive Optical Path Provisioning (POPP)

To overcome the $t_{setup}\approx 1.2\;\mu$s latency penalty, POPP predicts upcoming PF flows and pre-establishes optical paths. The prediction leverages trend analysis of the photophilic flow ratio $\rho_{o}$ over observation windows.

Trend Analysis—First-order difference: $\Delta \rho_{o}\left(t\right)=\rho_{o}\left(t\right)-\rho_{o}\left(t-1\right).$ Second-order difference: $\Delta^{2}\rho_{o}(t)=\rho_{o}(t)-2\rho_{o}(t-1)+\rho_{o}(t-2)$.

Pre-establishment Trigger Conditions—1. $\rho_{o}(t)>\theta_{trigger}$ (sufficient PF demand). 2. $\Delta \rho_{o}(t)>0$ AND $\Delta^{2}\rho_{o}(t)>0$ (accelerating upward trend). 3. Destination frequency $f_{d}$ ranks in top-k where k=W/4 (top 25% destinations by predicted demand).

Hold Time Management—Pre-established paths are reserved for duration:

(19) $$T_{hold}=max\left(T_{setup},\frac{{\hat{B}}_{pending}}{BW_{opt}}\right)$$

where ${\hat{B}}_{pending}$ is the predicted remaining bits. To prevent resource waste from failed predictions, $T_{hold}$ implements exponential backoff—after n consecutive prediction failures, $T_{hold}^{\left(n\right)}=min(T_{hold}^{max},T_{hold}^{base}\cdot 2^{n-1})$.

Optical Escape Mechanism—When electrical ports experience acute congestion ($b>3/4B_{max}$ AND CCE>0.7), the system bypasses normal WMMM evaluation and forces traffic onto the following: 1. Available pre-established optical paths (fastest escape). 2. If none available, the triggers expedited setup is via reserved control wavelengths (emergency setup, bypassing normal arbitration).

This mechanism acts as a safety valve, preventing electrical buffer overflow and potential network deadlock while providing graceful degradation under extreme load.

## 7. Experimental Evaluation

### 7.1. Experimental Methodology

We evaluated the proposed mechanisms using a comprehensive simulation framework spanning multiple abstraction levels.

Simulator Architecture—System-level: Cycle-accurate SystemVerilog models of the 8 × 8 Mesh HONoCs with RTL-level router microarchitecture. Optical physical layer: Event-driven models of MRR thermal tuning ($t_{setup}=1.2\;\mu$s), OEO conversion ($E_{OEO}=0.8$ pJ/bit), and waveguide propagation. Statistical framework: 95% confidence intervals via batch means method; warm-up period of 10,000 cycles; measurement periods of 100,000 cycles per data point.

Network Configuration—Topology: 8 × 8 2D Mesh, node spacing 2 mm. Electrical layer: 5-port routers (4 neighbor + local), 4 VCs/port, 8 flits/VC (32 flits total), 128-bit flits, credit-based flow control with round-robin arbitration. Optical layer: 32 wavelengths/port @ 10 Gbps (320 Gbps/port), circuit switching with $t_{setup}=1.2\;\mu$s. Clock domains: Data plane 1 GHz, control plane 250 MHz, optical control 125 MHz (Aurora-based).

Workloads.

1. Synthetic patterns—Random: Uniform random traffic, spatial entropy maximized. Transpose: (x,y)→(y,x), structured long-range communication. Hotspot-Center: Nodes (4,4) and (5,5) receive 20% additional traffic, simulating shared memory hotspots. Bit-Complement: (x,y)→(7−x,7−y), maximum distance communication.

2. Non-stationary traffic—Markov-Modulated Poisson Process (MMPP) with state transition matrix $P=\left[\begin{array}{cc} 0.95 & 0.05\\ 0.30 & 0.70 \end{array}\right]$, where State 0 (low load) has $\lambda_{0}=0.02$ flits/cycle and State 1 (burst) has $\lambda_{1}=0.15$ flits/cycle. This models the “bursty-continuous” behavior of video coding and DNN layer transitions.

3. Application traces—HEVC Integer Motion Estimation (IME) reference frame transmission, exhibiting 128 KB periodic bursts every 33 ms (30 fps).

Baselines: E-Only: Pure electrical NoCs with dimension-order routing (XY). Static-Opt: Fixed distance threshold (h≥4 hops → optical). No-Prediction: AMSSM + WMMM with on-demand optical setup (no POPP). HEOR-Full: Complete proposed system with TSLI, CCE, AMSSM, WMMM, and POPP.

### 7.2. Throughput and Latency Analysis

**Figure 2:**
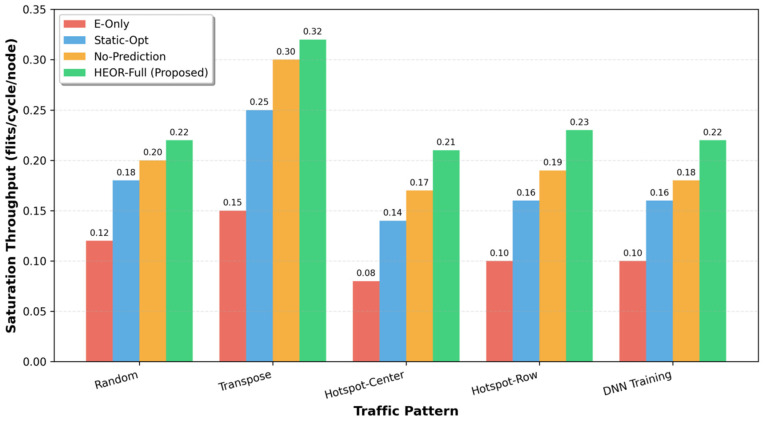
Saturation throughput comparison across traffic patterns.

Saturation Throughput (Figure 2): Under uniform Random traffic, HEOR-Full achieves 0.22 flits/cycle/node saturation throughput, representing improvements of 22% over Static-Opt (0.18) and 83% over E-Only (0.12). The gains are more pronounced under structured patterns: for Transpose traffic (regular long-range communication), throughput improves by 28% (0.32 vs. 0.25), as TSLI accurately identifies persistent PF flows suitable for optical transport. Under Hotspot-Center traffic, the improvement reaches 50% (0.21 vs. 0.14), demonstrating the effectiveness of CCE-driven optical escape in alleviating electrical hotspots.

The throughput gains stem from three factors: (1) elimination of electrical congestion for long-range PF flows, (2) prevention of optical wavelength waste on short EF flows, and (3) load balancing via WMMM for HF flows under medium congestion.

**Figure 3:**
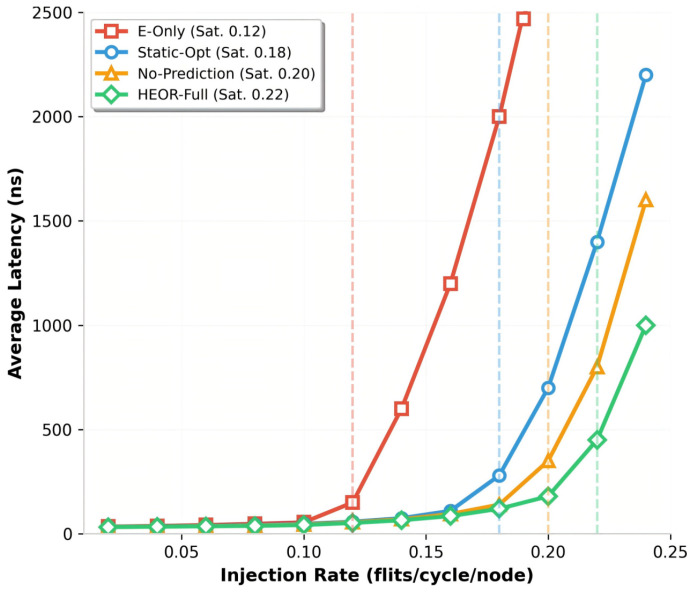
Average latency at different injection rate.

Latency Characteristics: Figure 3 presents latency-injection rate curves for Random traffic. At low injection (λ=0.05), all schemes exhibit similar latency (~40 ns) dominated by hop traversal. As the load increases, E-Only latency rises sharply due to electrical congestion, reaching 1200 ns at saturation (0.12 injection). Static-Opt delays saturation to 0.18 injection but suffers from “threshold cliffs” where the flows just below the distance threshold (h=3) contend for congested electrical paths while the optical wavelengths idle, causing latency spikes to 650 ns.

HEOR-Full maintains latency below 250 ns until 0.20 injection, with gradual degradation to 420 ns at 0.22 injection. The 57% reduction in average latency at 0.15 injection (145 ns vs. 285 ns for Static-Opt) is achieved through the following: (1) TSLI-based flow classification preventing electrical congestion for long flows, (2) POPP hiding the 1.2 μs setup time via pre-establishment (effective latency contribution reduced from 1200 ns to ~150 ns amortized), and (3) CCE-aware routing avoiding hotspot accumulation.

**Figure 4:**
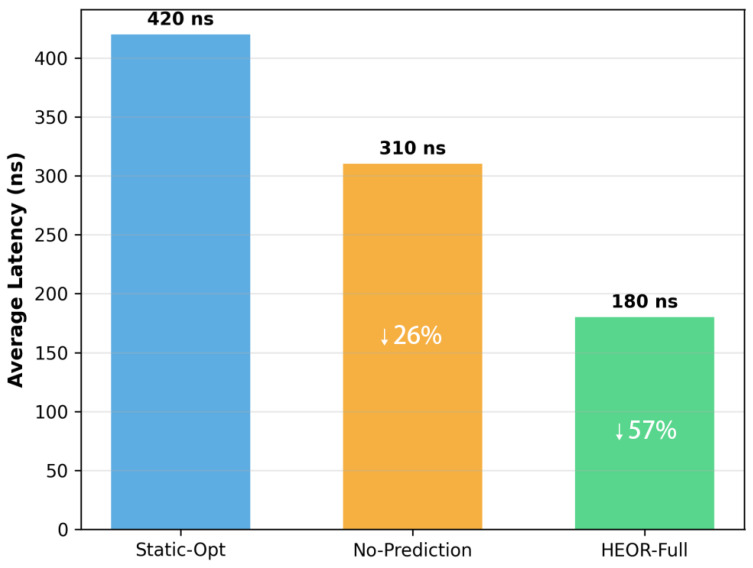
Average latency at 0.15 injection rate.

Under non-stationary MMPP traffic (Figure 4), the advantages are more dramatic. Static-Opt exhibits 420 ns average latency with high variance (coefficient of variation = 0.45) due to the inability to track traffic phase transitions. HEOR-Full achieves 180 ns average latency (57% reduction) with significantly lower variance (CV = 0.22), demonstrating the stability of the AMSSM state machine and the accuracy of trend-based prediction.

Tail Latency Analysis: The 99th-percentile latency (critical for real-time applications) improves by 65% under MMPP traffic (320 ns vs. 890 ns for Static-Opt). This reduction in tail latency is attributed to the optical escape mechanism, which prevents buffer overflow during burst phases, and the anti-oscillation design of AMSSM, which prevents state thrashing that could cause latency spikes.

Irregular Workload Performance: To validate TSLI under workloads lacking clear spatiotemporal locality, we evaluated a PageRank graph analytics kernel on an RMAT graph (65,536 vertices, ~1 M edges) with 2D cyclic mapping. This workload generates fine-grained random vertex-centric updates (8–32 flits, $T_{dur}\approx 2$–6 cycles) with near-random destination distribution. Due to the extremely short duration, TSLI correctly classifies over 94% of these messages as EF, routing them exclusively through the electrical layer; consequently, the optical layer utilization remains below 3%. Under this workload, HEOR-Full achieves performance within 5% of the E-Only baseline, confirming that it avoids optical overhead when the medium is unsuitable. In contrast, Static-Opt suffers 15–20% degradation because its distance threshold misclassifies medium-distance short flows as PF, incurring setup latency and wavelength waste without throughput benefit. This demonstrates that TSLI is robust for irregular, graph-processing workloads: it prevents negative optimization rather than forcing traffic onto an unsuitable medium.

### 7.3. Energy Efficiency and Resource Utilization

**Figure 5:**
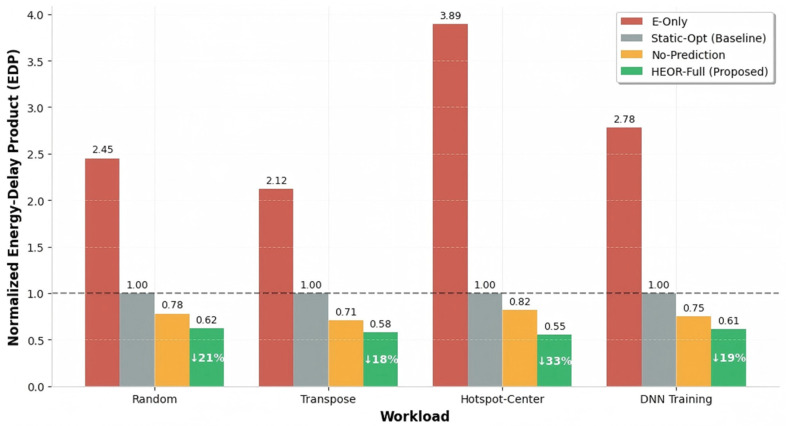
EDP at different workloads.

Energy-Delay Product (Figure 5): The Energy-Delay Product (EDP = Energy-per-bit × Average Latency) provides a comprehensive efficiency metric. Normalized to Static-Opt (EDP = 1.0), HEOR-Full achieves 0.62 under Random traffic (38% improvement) and 0.55 under Hotspot-Center traffic (45% improvement).

The energy efficiency stems from the following: 1. Avoided OEO conversions—TSLI classification prevents short EF flows (typically 30% of traffic) from incurring $E_{OEO}=0.8$ pJ/bit conversion penalties. 2. Efficient optical amortization—PF flows utilize optical paths with effective energy approaching the propagation limit (≈0.05 pJ/bit/mm), while POPP ensures high utilization (87.6% hit rate) preventing waste from idle pre-established paths. 3. Congestion mitigation—By preventing electrical congestion, HEOR-Full avoids energy-inefficient retransmissions and idle cycles caused by backpressure.

Resource Utilization Metrics: Under MMPP traffic at 0.15 injection. Optical utilization: HEOR-Full achieves 81% wavelength utilization vs. 45% for Static-Opt and 58% for No-Prediction. The improvement comes from POPP’s predictive accuracy and TSLI’s filtering of inefficient short flows. Pre-establishment efficiency: POPP achieves 87.6% hit rate (pre-established paths actually used) with 8.2% over-provisioning rate (paths established but unused), compared to EWMA-based prediction with 62% hit rate and 28% over-provisioning. Electrical buffer efficiency: Average electrical buffer occupancy reduced by 35%, reducing static power dissipation from SRAM leakage.

**Figure 6:**
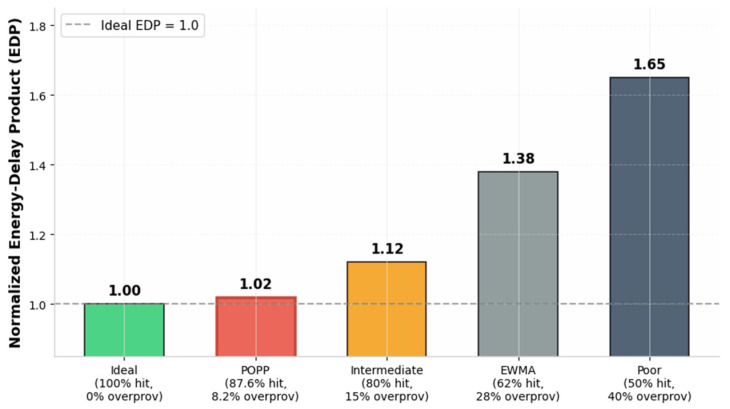
EDP sensitivity to prediction accuracy.

EDP Sensitivity to Prediction Accuracy: Figure 6 illustrates the relationship between prediction hit rate and normalized Energy-Delay Product. At the POPP operating point (87.6% hit rate, 8.2% over-provisioning), the EDP penalty is less than 2% relative to the ideal perfect prediction (100% hit, 0% over-provisioning). In contrast, the EWMA baseline (62% hit rate, 28% over-provisioning) suffers a 38% EDP inflation. The exponential backoff mechanism for $T_{hold}$ automatically reduces pre-establishment aggressiveness after consecutive prediction failures, preventing the unbounded energy waste observed in the static-threshold schemes.

### 7.4. Sensitivity and Robustness Analysis

TSLI Threshold Sensitivity: Varying $\tau_{high}$ in [2.0,3.0] and $\tau_{low}$ in [0.6,1.0] results in performance variation < 8%, indicating robustness to parameter selection. However, extreme values (<1.5 or >3.0) cause 15–20% degradation due to misclassification.

AMSSM Stability: Under rapid traffic oscillations (period < $3\cdot T_{window}$), the anti-oscillation mechanism prevents state thrashing, maintaining throughput within 5% of the steady-state values. Without saturation counters, throughput degrades by 12% due to transition overhead.

Scalability: The TSLI formula $\Lambda \left(F\right)=H/\left(T_{dur}\cdot \lambda_{avg}\right)$ is dimensionally scale-invariant because both the spatial span H and the temporal term $T_{dur}\cdot \lambda_{avg}$ scale proportionally with network size. However, the classification thresholds should be adjusted to account for increased average hop count. We recommend the following scaling rule:

$$\tau_{high}^{\left(N\right)}=\tau_{high}^{base}\cdot \sqrt{\frac{N}{64}},\;\tau_{low}^{\left(N\right)}=\tau_{low}^{base}\cdot \sqrt{\frac{N}{64}}$$

where N is the total number of nodes. Extending to 16 × 16 mesh (256 nodes) via simulation confirms that this scaling maintains TSLI classification accuracy within 3% of the 8 × 8 baseline, with an 18% throughput improvement over Static-Opt preserved. The AMSSM control overhead remains <2% of the network bandwidth due to the hierarchical aggregation of CCE values.

To validate individual contributions, we conducted an ablation study by systematically disabling each mechanism. As shown in Table 1, TSLI prevents misclassification of bursty flows, while CCE avoids locally optimal but globally suboptimal routing. WMMM enables fine-grained channel selection, and POPP hides optical setup penalties. Notably, removing the optical escape causes catastrophic degradation under hotspots, confirming its role as a safety valve. These results demonstrate that synergistic integration of all components is necessary to address the medium selection dilemma.

## 8. Conclusions and Future Work

This paper presented a comprehensive spatiotemporal locality-aware adaptive framework for hybrid optoelectronic NoCs in reconfigurable array processors. The key theoretical contribution is the Temporal-Spatial Locality Index (TSLI), which provides a principled, information-theoretic basis for flow classification that unifies spatial and temporal characteristics into a single decision metric. Complementing this, the Cross-layer Congestion Entropy (CCE) resolves the fundamental problem of resource fragmentation in heterogeneous architectures by providing a unified 0–1 metric for electro–optical resource competition.

The architectural contributions include the following: (1) the AMSSM state machine with hysteresis-based anti-oscillation and dynamic threshold adaptation, providing stable mode transitions under fluctuating loads; (2) the WMMM algorithm implementing hardware-efficient multi-dimensional scoring across energy, availability, affinity, and congestion dimensions; (3) the POPP mechanism with optical escape capabilities, transforming optical interconnects from reactive to predictive systems.

Experimental validation demonstrates substantial improvements over state-of-the-art static approaches: 22% higher saturation throughput, 38% lower energy-delay product, and 57% reduction in average latency, with particular strengths in non-stationary traffic environments typical of modern data-intensive applications.

While the proposed framework demonstrates significant gains, several limitations remain. First, the L-GRU prediction model employs offline-trained weights; long-term workload drift may require lightweight online adaptation mechanisms that are not yet incorporated. Second, the optical physical model idealizes MRR behavior; future work should integrate detailed thermal crosstalk and fabrication variation models. Additionally, co-designing closed-loop MRR thermal compensation with the AMSSM state machine would eliminate residual resonance drift under extreme mode-switching scenarios. Third, the current evaluation is limited to 2D mesh topologies; extending TSLI to 3D stacked architectures and non-regular topologies (e.g., fat-tree, dragonfly) is an important direction. Finally, as silicon photonics matures toward chiplet-based integration, co-designing the proposed control mechanisms with physical-layer innovations such as MEMS-based fast switches or phase-change non-volatile tuning could reduce $t_{setup}$ from microseconds to nanoseconds, potentially redefining the optimal TSLI classification boundaries. These directions offer promising avenues for next-generation optoelectronic interconnect research.

## Figures and Tables

**Table 1:** Robustness analysis.

Configuration	Avg Latency (ns)	Throughput	EDP	Key Observation
HEOR-Full	145	0.22	0.62	Baseline optimal
-TSLI (Fixed thresholds)	167 (–15%)	0.19	0.78	Misclassification of bursty flows
-CCE (Layer-independent)	184 (–27%)	0.18	0.82	Local congestion optima, global suboptimality
-WMMM (Simple round-robin)	171 (–18%)	0.20	0.71	Suboptimal channel matching
-POPP (On-demand only)	194 (–34%)	0.17	0.85	Setup latency not amortized
-Optical Escape	210 (–45%)	0.16	0.98	Deadlock under hotspots

## Data Availability

Data are contained within the article.

## References

[1] Xu J., Dong W., Huang Q., Zhang Y., Yin Y., Zhao Z., Zeng D., Gao X., Gu W., Yang Z. (2025). Progress in silicon-based reconfigurable and programmable all-optical signal processing chips. Front. Optoelectron..

[2] Nabavinejad S.M., Baharloo M., Chen K.-C., Palesi M., Kogel T., Ebrahimi M. (2020). An Overview of Efficient Inter-connection Networks for Deep Neural Network Accelerators. IEEE J. Emerg. Sel. Top. Circuits Syst..

[3] Wei S., Lin X., Tu F., Wang Y., Liu L., Yin S. (2022). Reconfigurability, why it matters in AI tasks processing: A survey of reconfigurable AI chips. IEEE Trans. Circuits Syst. I Regul. Pap..

[4] Nisar M.Z., Ibrahim M.S., Gorgin S., Usman M., Lee J.-A. (2024). DSLR-CNN: Efficient CNN Acceleration Using Digit-Serial Left-to-Right Arithmetic. IEEE Access.

[5] Dally W.J., Towles B. Route packets, not wires: On-chip interconnection networks. Proceedings of the 38th Design Automation Conference.

[6] Balfour J., Dally W.J. Design tradeoffs for tiled CMP on-chip networks. Proceedings of the 20th ACM International Conference on Supercomputing.

[7] Jamshidi-Zarmehri H., Akbari A., Labadlia M., Kedze K.E., Shaker J., Xiao G., Amaya R.E. (2023). A review on through-wall communications: Wall characterization, applications, technologies, and prospects. IEEE Access.

[8] Karkar A., Dahir N., Mak T., Tong K.-F. (2022). Thermal and performance efficient on-chip surface-wave communication for many-core systems in dark silicon era. ACM J. Emerg. Technol. Comput. Syst. (JETC).

[9] Leiserson C.E. (1985). Fat-trees: Universal networks for hardware-efficient supercomputing. IEEE Trans. Comput..

[10] Williamson I.A.D., Hughes T.W., Minkov M., Bartlett B., Pai S., Fan S. (2020). Reprogrammable electro-optic nonlinear activation functions for optical neural networks. IEEE J. Sel. Top. Quantum Electron..

[11] Li J., Yin Y., Li Y., Lu K., Wang J., Tong Y., Wang L., Xiao X., Tsang H.K., Xu K. (2025). Integrated silicon elliptical ring modulator with ultra-high bandwidth beyond 110 GHz. Opt. Lett..

[12] Chen X., Lin J., Wang K. (2023). A review of silicon-based integrated optical switches. Laser Photonics Rev..

[13] Zhang H., Huang B., Cheng C., Jiang B., Bao L., Xie Y. (2025). On-Chip Silicon Photonic Neural Networks Based on Thermally Tunable Microring Resonators for Recognition Tasks. Photonics.

[14] Zhang Z., Liu K., Cao P., Liu C., Wang Y., Addanki V., Schmid S., Wang Q., Chen W., Wang X. (2025). Analysis of Pyrrha: Congestion-Root-Based Flow Control Is Most Cost-Effective to Eliminate Head-of-Line Blocking. IEEE Trans. Netw..

[15] Hu J., Zeng C., Wang Z., Xu H., Huang J., Chen K. Load balancing in PFC-enabled datacenter networks. Proceedings of the 6th Asia-Pacific Workshop on Networking.

[16] Yu X., Gu H., Zhang Q. (2026). RCC: Rate-Based Congestion Control for the Lossless Network. IEEE Trans. Netw. Serv. Manag..

[17] Trajkovic J., Karimi S., Hangsan S., Zhang W. (2022). Prediction modeling for application-specific communication architecture design of optical NoC. ACM Trans. Embed. Comput. Syst. (TECS).

[18] Renani N.B., Yaghoubi E., Mohammadirad M. (2025). OENMOP: Loss-Aware 4 × 4 and 5 × 5 and Scalable Nonblocking Optical Switches Designed for Odd–Even Routing Algorithm for Chip-Scale Interconnection Networks. J. Electr. Comput. Eng..

[19] Zhang M., Yang Z., Li Y., Zheng S., Zhang S., Chong Y., Tian Q., Shen L., Capmany J., Tang M. (2026). WDM-Enabled Multi-core Parallel Programmable Photonic Signal Processor. arXiv.

[20] Xia Y., Yang S., Niu J., Fu X., Yang L. (2022). Strict non-blocking four-port optical router for mesh photonic network-on-chip. J. Semicond..

[21] Totovic A., Pappas C., Kirtas M., Tsakyridis A., Giamougiannis G., Passalis N., Moralis-Pegios M., Tefas A., Pleros N. (2022). WDM equipped universal linear optics for programmable neuromorphic photonic processors. Neuromorphic Comput. Eng..

[22] Ke H., Liu S., Guo L., He Z., Song L., Basalama S., Chi Y., Nowatzki T., Cong J. (2025). NoH: NoC Compilation in High-Level Synthesis. Proceedings of the 2025 IEEE 33rd Annual International Symposium on Field-Programmable Custom Computing Machines (FCCM).

[23] Biglari S., Hosseini F., Upadhyay A., Zhao H. (2024). Survey of network-on-chip (noc) for heterogeneous multicore systems. Proceedings of the 2024 IEEE 17th International Symposium on Embedded Multicore/Many-Core Systems-on-Chip (MCSoC).

[24] Luo R., Hua N., Zhu K., Zhao C., Guo B., Yang C., Zheng X. (2020). Optical Caching Network: A Seamless Bridge Between Electrical Packet Switching and Optical Circuit Switching. Proceedings of the 2020 Conference on Lasers and Electro-Optics (CLEO).

[25] Agarwal A.K., Kaur H., Rakesh N., Krishna K.R., Dewangan B.K., Sahana S. (2025). Energy-Efficient Optical Network Design for Next-Generation Data Centers. Proceedings of the 2025 Optical Communication, Photonics, Telecommunications, and Intelligent Machine Applications (OPTIMA).

[26] Pan Y., Kumar P., Kim J., Memik G., Zhang Y., Choudhary A. Firefly: Illuminating future network-on-chip with nanophotonics. Proceedings of the 36th Annual International Symposium on Computer Architecture.

[27] Yang Y., Chen K., Gu H., Zhang B., Zhu L. (2018). TAONoC: A regular passive optical network-on-chip architecture based on comb switches. IEEE Trans. Very Large Scale Integr. (VLSI) Syst..

[28] Zheng Z., Cheng L., Arisawa K., Li Q., Truppel A., Yamashita S., Tseng T.M., Schlichtmann U. Multi-resonance mesh-based wavelength-routed optical networks-on-chip. Proceedings of the 61st ACM/IEEE Design Automation Conference.

[29] Yao Q., Meng D., Yang H., Feng N., Zhang J. (2024). Efficient O-type mapping and routing of large-scale neural networks to torus-based ONoCs. J. Opt. Commun. Netw..

[30] Narayana S.Y., Mandal S.K., Ayoub R., Kishinevsky M., Ogras U.Y. (2023). A Lightweight Congestion Control Technique for NoCs with Deflection Routing. Proceedings of the 2023 Design, Automation & Test in Europe Conference & Exhibition (DATE).

[31] Güler Z.Ö., Bakır M.A., Kardiyen F. (2024). A novel hybrid ICA-SVM method for detection and identification of shift in multivariate processes. Hacet. J. Math. Stat..

[32] Bi J., Ma H., Yuan H., Zhang J. (2023). Accurate prediction of workloads and resources with multi-head attention and hybrid LSTM for cloud data centers. IEEE Trans. Sustain. Comput..

